# Analysing the support mechanisms of the vaginal ring pessary on supine and upright MRI

**DOI:** 10.1038/s41598-024-81985-9

**Published:** 2024-12-28

**Authors:** Frieda van den Noort, I. de Alba Alvarez, A. van der Steen, A. D. Smelt, F. F. J. Simonis, A. T. M. Grob

**Affiliations:** 1 Multi-Modality Medical Imaging (M3I), TechMed Centre, University of Twente, Technohal 2384,Drienerolaan 5, Enschede, 7522NB The Netherlands; 2 Department of Gynecology, Ziekenhuisgroep Twente, Hengelo/Almelo, The Netherlands; 3 Magnetic Detection and Imaging (MD&I), TechMed Centre, University of Twente, Enschede, The Netherlands

**Keywords:** Outcomes research, Magnetic resonance imaging

## Abstract

Vaginal pessaries have been used for millennia to alleviate symptoms of pelvic organ prolapse (POP). Despite their long-standing use, the success rate of pessary treatment is approximately 60%, and the underlying mechanisms of support are not well understood. This study aims to investigate three previously proposed hypotheses regarding the support mechanisms of pessaries, utilizing supine and upright magnetic resonance imaging (MRI): (1) support by bony structures, (2) support by levator ani muscles (LAM), and (3) the uterus keeping the pessary in place by acting as a lever. Thirty POP patients, with a successful pessary fit for more than 3 months, underwent MRI scans in both upright and supine position. The position of the pelvic bony structures, LAM, uterus, and pessary were analysed by placing corresponding points on these structures and comparing the differences between supine and upright. Also, the angle of the pessary with the horizontal plane of the pelvic inclination corrections system was analysed. Principal component analysis was applied to evaluate how the positions of the LAM and the pessary changed between the two positions. The lowest point of the pessary descends below the lowest point of the bony structures in upright position, disproving the first hypothesis. The pessary significantly descends towards the LAM from supine to upright, but does not drop below it, strengthening the second hypothesis. The pessary angle is below 90° in upright position, the uterus is positioned in the pessary centre and remains constant from supine to upright, strengthening the third hypothesis.

## Introduction

Vaginal pessaries are used to alleviate symptoms of pelvic organ prolapse (POP) and/or urinary incontinence^1^. The use of pessaries dates back millennia^1^, with historical evidence of a bronze, donut-shaped pessary being used by the Romans, bearing a striking resemblance to the modern donut pessary^2^. To this day, the pessary remains a popular conservative treatment option for POP. However, their success rate, defined as the percentage of patients who continue wearing a pessary at 3 months follow-up, is only 63% ^3^. The primary reason for unsuccessful use is dislodgement, suggesting insufficient support of the pessary.

Throughout history, numerous pessary shapes and types have been developed and tested, with the ring pessary (with or without support) currently being the most used pessary^1,3^. The development and adaptation of these pessary types has been mainly based on an informed trial and error process, leading to new shapes, e.g. Gellhorn and cube, each with their specific use-cases. However, the understanding how a pessary is supported to stay in place or why a pessary relieves symptoms is still lacking.

Recently, the advent of 3D printing enabled the creation of personalized pessaries, which sparked a new interest in understanding the functional mechanisms of pessaries^4^. Several studies have investigated the feasibility of personalized pessaries^5–9^, with several also reporting successful long-term use of the personalized pessary^7–9^. To come to a new pessary-design the studies used general assumptions about the pessary, patient feedback and ultrasound imaging^5,7–9^. While this approach adds value to the personalization of pessaries, it does not provide a general insight into pessary support.

In this study, we aim to gain a more fundamental understanding of the support mechanisms for pessaries, with a particular focus on the ring pessary, as it is the most used type^1,3^. Several hypotheses have been proposed in the literature regarding the supporting structures of the ring pessary, with the possibility that the answer is found in a combination of (parts of) these theories/hypotheses.

1. The pessary is supported by a bony pelvic structure, likely the pubic symphysis^10,11^. This hypothesis is based on the general supportive role of bony structures in the body.
2. The pessary is supported by the levator ani muscle (LAM)^12–16^. This hypothesis is supported by the fact that damage to the LAM is a significant factor in the failure of pessary fitting. Also, the ratio of hiatal area to ring pessary size is predictive for pessary success^14^.
3. The pessary rests in the posterior fornix and is thus held in place by the uterus, which acts as a lever to secure the pessary^11,15,17^. This hypothesis is supported by the higher incidence of unsuccessful pessary fits in women who have undergone a hysterectomy^3^.

To test the above-mentioned hypotheses on pessary support, the bony and soft tissue structures of the full pelvis should be analysed, which is possible by means of Magnetic Resonance Imaging (MRI), which has a larger field of view than ultrasound. Two recent studies^15,16^ studied pessaries in situ using MRI, however both studies used supine MRI. Since the pessary is worn in upright position and dislodgement also mainly occurs in upright position, combined with recent literature on the difference in pelvic anatomy in supine and upright position^18–20^, we hypothesize that an accurate assessment of the pessary support mechanism can only be done with the patient in upright position.

In this study, we evaluate the validity of the support hypotheses by analysing upright and supine MRI scans of POP patients, with a successful ring pessary fit. We aim to identify the differences in pessary position between the supine and upright position relative to key anatomical structures. Using this approach, we work towards a better understanding of the ring pessary support within POP patients and aim to provide more clarity on the validity of existing pessary support hypotheses.

## Methods

### Population

In this prospective study, 30 POP patients with a successful ring pessary fit (defined as continued pessary use for a minimum of three months) were included. These women were recruited from the gynecology department of the Ziekenhuisgroep Twente (ZGT) hospital in Hengelo, the Netherlands. The study received approval from the medical ethics committee and registered as NL74061.091.20. The methods were caried out following the guidelines of the ethics committee and all study participants gave written informed consent. All women presented primarily with POP symptoms and were clinically assessed to have a POP-Q stage ≥ 2 of the anterior and/or apical compartment. To study a homogenous group, only patients with ring pessaries, with and without support membrane, were included. All women included in the study were aged 18 years or older. Exclusion criteria were an inability to stand unassisted for 20 min, ineligibility for MRI based on an MRI safety checklist, or a jeans size of ≥ 52 (EU) or ≥ 22 (US), due to the limited circumference of the MRI coil.

### MRI examination

MRI scans of the women were acquired in both upright and supine position with the pessary in situ. Participants were instructed not to drink for one hour prior to the scan and had to empty their bladder within 15 min before the procedure. Imaging was conducted using a tiltable 0.25T MRI scanner (G-Scan Brio; Esaote S.p.A., Genoa, Italy) equipped with a dedicated multichannel spine coil. A 3D balanced steady state free precession (bSSFP) sequence was acquired (TE/TR: 4/8 ms, flip angle: 60°, reconstructed resolution: 0.49 × 0.49 × 0.49 mm^3^, FOV: 250 × 250 × 122 mm^3^ or 250 × 250 × 160 mm^3^, acquisition matrix 124 × 124 × 100, number signal averages 3, scan time: ± 5 min).

### Landmark annotation

We analysed the images by placing landmarks on both the supine and upright MRI scans using 3D Slicer (v5.2.1, www.slicer.org)^21^. First, we identified the position of the points that define the 3D pelvic inclination coordinate system (PICS)^22^, which provided the position of the bony landmarks and allowed us to analyse their position with respect to the pessary.

**Fig. 1:**
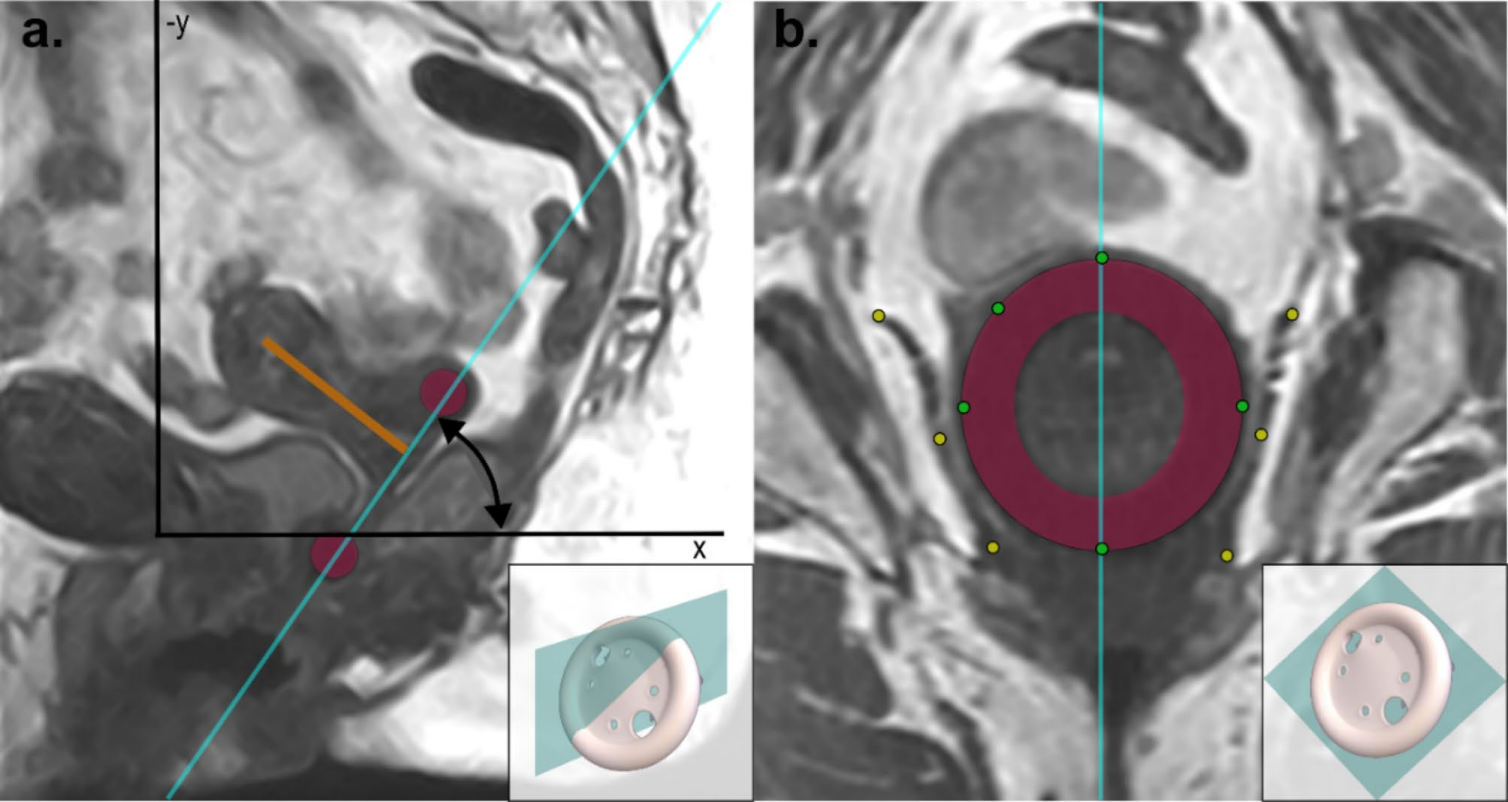
(a) A midsagittal view of a patient in an upright position with a pessary (depicted in pink) in situ. The endometrial line of the uterus is visualized (depicted in orange), and the position of the image slice shown in (b). Is indicated by the blue line. In the lower right corner, a pessary is depicted with a blue plane, illustrating its appearance on this midsagittal MRI slice. Also, the PICS coordinate system is visualized in black (z is not visualized since it is orthogonal to the image), the arch which arrow indicates how the angle of the pessary is calculated. (b) The MRI slice that cross-sects the pessary. The blue line indicates the position of the midsagittal slice depicted in (a). The green points mark the top, right, bottom, and left positions on the pessary, with an additional point between the top and left positions to capture its orientation. The yellow points mark the top, middle, and bottom positions of the levator ani muscle on both the left and right sides. In the lower right corner, a pessary is depicted with a blue plane, illustrating its appearance on this MRI slice.

Next, the position of the LAM relative to the pessary was analysed in a cross-sectional view of the pessary (see Fig. 1b). In this view we annotated the top, bottom, left and right sides of the pessary and an additional pessary point between the top and the left point, enabling us to fit the pessary shape through the points for visualization. Additionally, we identified the top and bottom points of both the left and right side of the LAM and added an extra point in the middle to accurately trace the LAM shape.

Finally, to investigate the role of the uterus in pessary support, we identified (1) the orientation of the uterus according to the anteverted or retroverted position on the patient scans without a pessary, and (2) the endometrial line (see Fig. 1a) by placing a point at the top and the bottom of the endometrium. This was performed in 3D to capture out-of-sagittal plane rotation of the uterus.

### Analysis of landmark position

The identified landmark points were imported into Matlab (2022b, Natick, Massachusetts) for further analysis. The PICS points enable the correction of patient tilt during scanning, ensuring that all points are positioned within a consistent coordinate system. This allows for the comparison of data from upright and supine positions within the same reference frame. The x (anterior to posterior), y (cranial to caudal), and z (left to right) axes are defined relative to the patient, independent of their position or orientation within the scanner (see Fig. 1)^22,23^. The lowest point of the pessary was analysed, and the angle and mid-point of the pessaries were calculated relative to the PICS. These parameters were compared between the supine and upright positions to assess any differences (see Fig. 1a).

The changes in the position of the LAM relative to the pessary between upright and supine positions were investigated using principal component analysis (PCA) on the pessary and LAM points (Fig. 1b) in 2D, in the MRI slice in which they were identified. This approach is analogous to the levator plate shape analysis proposed by Schmidt et al.^24^ though different input points were used. The advantage of PCA lies in its ability to capture the largest shape variation within a dataset using only a few principal components (PCs). Each shape in the dataset (in this case, represented by five pessary points and six LAM points) is characterized by a single value on these PCs, facilitating statistical analysis between supine and upright positions. For PCA to be effective, the data must be normalized by aligning the individual datasets to overlap (by translating, rotating, and scaling the points). This ensures that the shape variation captured by PCA is the variation of interest. In this case, normalization was achieved by aligning the left and right pessary points of all patients to the same position.

Finally, the endometrial line of the uterus was analysed relative to the pessary. The data were normalized by aligning the pessary points and endometrial lines so that the left and right sides of the pessaries overlap, ensuring that the pessary points are all in the same plane.

### Statistical analysis

Statistical analysis was performed in Matlab by applying the Wilcoxon signed rank test.

## Results

The median age of the 30 included women was 64.4 years old (range 42–79), body mass index (BMI) 25.9 (19–38) kg/m^2^ and parity 2 (1–6). 73% of the women (*n* = 22) were post-menopausal and 57% (*n* = 17) were sexually active. All patients had their uterus in situ, of whom six patients (20%) presented with a retroverted position.

**Fig. 2:**
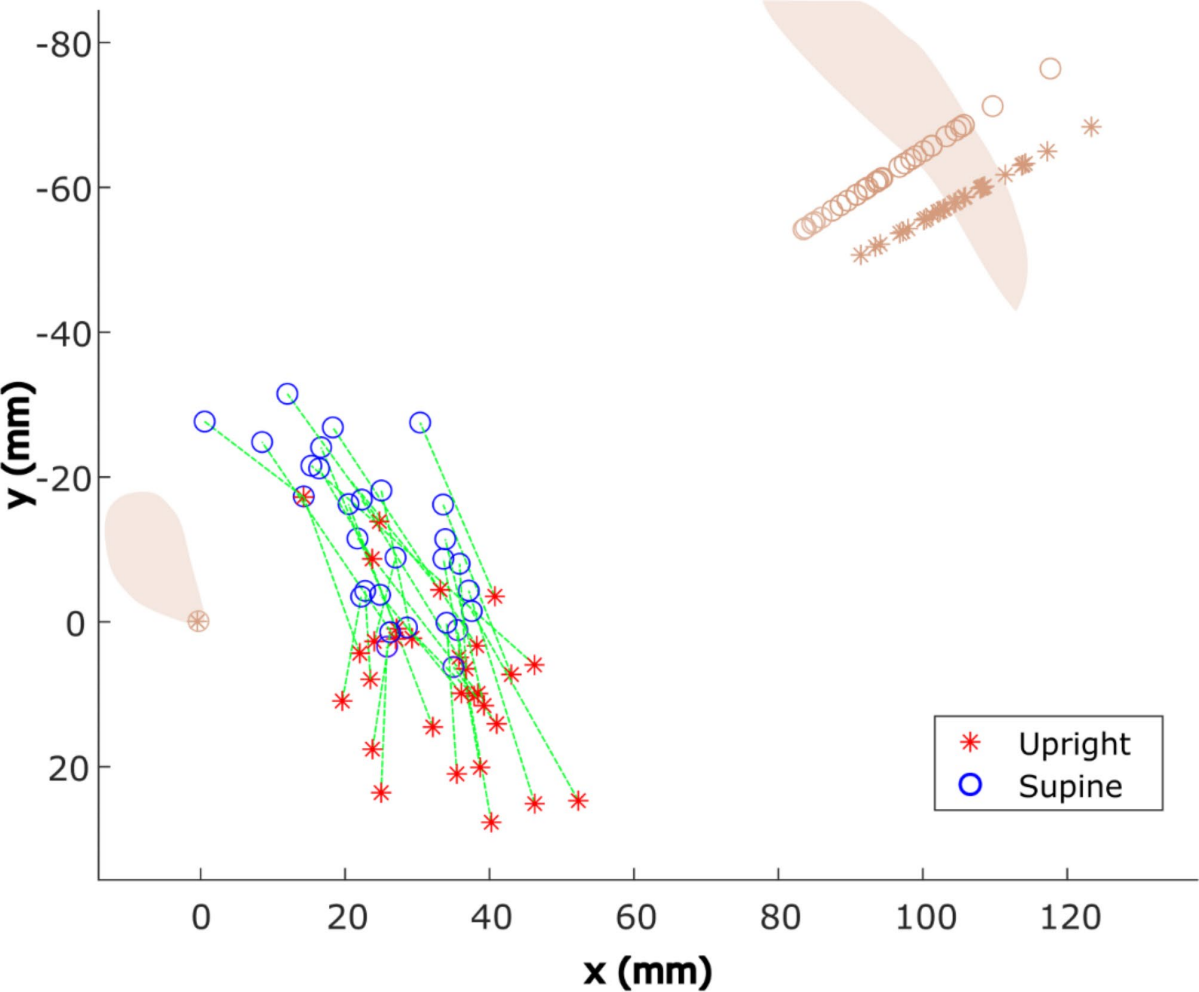
The position of the lowest point of the pessary in the midsagittal view is illustrated. Data for both supine (blue circles) and upright (red stars) positions are presented and connected per individual patient by green lines. For anatomical reference, the positions of the symphysis and the sacrococcygeal point are included (beige). Note that the symphysis points are the origin of the PICS coordinate system, while the position of the sacrococcygeal point differs between the supine and upright positions due to the varying angles (resp. 34° and 29°) observed between these postures^23^.

Table 1 presents the medians, minimum and maximum values of the lowest and mid-point of the pessary and the angle of the pessary in the midsagittal plane. The movements of the lowest and mid-point of the pessary from supine to upright show a significant caudal descent and the pessary gets significantly oriented towards posterior direction, as illustrated in Fig. 2. Regarding the pessary support hypotheses, it is important to note that the y position of almost all pessaries is positive, indicating a position below the pubic bone when in upright position. The lowest point of the pessary shows a total minimal, maximal, and median movement of 10.3 mm, 39.8 mm, and 23.4 mm, respectively, within a single patient between supine and upright positions. Even though we found the angle of the pessary to be significantly larger in the supine position, a considerable variation exists among patients. In ten patients, an opposite trend was noted, where the pessary angle became smaller in supine position. Within the total group, for nine patients, the pessary angle remained relatively unchanged, with a difference of less than 5°. Conversely, in ten patients, the difference between the two angle measurements exceeded 10°, with one patient even presenting a 33° difference.

**Table 1:** This table presents the medians, minimum, and maximum values of the lowest and mid-point of the pessary and the angle of the pessary with respect to PICS reference frame (noting that in PICS, negative y is defined cranially) for both supine and upright positions.

	Supine median (min–max)	Upright median (min–max)	Significance *p*-value
x position lowest point pessary (mm) (anterior-posterior)	24.9 (0.6 to 37.5)	35.3 (19.6 to 52.3)	< 0.001
y position lowest point pessary (mm) (cranial-caudal)	− 11.5 (− 31.5 to 6.2)	10.1 (− 13.9 to 27.7)	< 0.001
z position lowest point pessary (mm) (left-right)	− 2.0 (− 6.8 to 11.1)	− 0.3 (− 7.5 to 5.9)	0.81
x position mid-point pessary(mm) (anterior-posterior)	42.7 (25.7 to 61.5)	52.4 (33.5 to 68.1)	< 0.001
y position mid-point pessary (mm) (cranial-caudal)	− 41.2 (− 55.5 to − 22.9)	− 19.2 (− 37.7 to − 4.4)	< 0.001
z position mid-point pessary (mm) (left-right)	− 1.9 (− 8.8 to 9.4)	− 0.9 (− 8.7 to 4.7)	0.72
Angle pessary to PICS in midsagittal view (°)	61.7 (28.4 to 100.1)	56.3 (38.5 to 85.6)	0.024

For the visualization of the orientation of the x, y and z values we refer to Fig. 1a, the orientation of the x and y axis are visualized here. The z axis is perpendicular to this view. Additionally, the p-values obtained by the Wilcoxon signed-rank test are included.

**Fig. 3:**
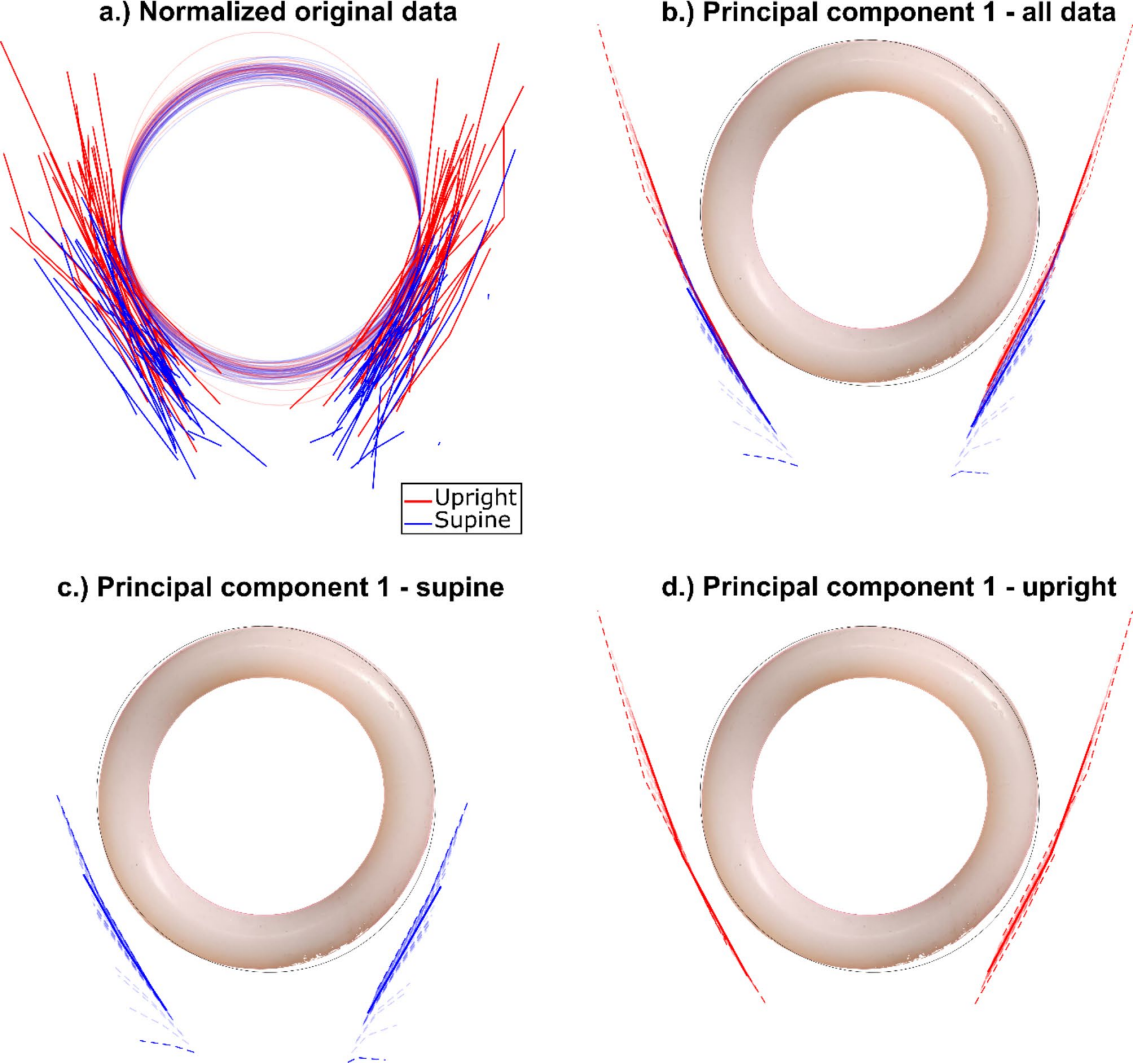
The point data of the pessary and levator ani muscle (LAM) for all patients is presented, for visualisation purposes lines are drawn through the points. (a) Displays the normalized original data. (b–d) Show the first principal component for the LAM relative to the pessary, resp. supine and upright in a single subfigure, only supine and only upright. Each individual patient is represented by a set of opaque lines. Non-opaque lines denote the extreme principal component values (dotted lines) and the average principal component value (bold line).

Applying PCA to the normalized point data of the pessary and LAM (see Fig. 3a) resulted in a decomposition where the amount of shape variance explained by the first six principal components (PC) were 72.2%, 9.0%, 7.8%, 5.4%, 2.6%, and 1.1%, respectively, with the remaining components accounting for less than 1% each. Among these, only the first PC showed a significant difference between supine and upright positions (*p* < 0.001). The data for the patients on this first PC are shown in Fig. 3b–d. This first PC captures length variation within the LAM as well as how close the pessary is to the LAM. In the supine position, the LAM is smaller, likely because the pessary is positioned more anteriorly, resulting in a more anterior cross-section of the LAM, which is smaller in this region. Additionally, the pessary angle is steeper in the supine position, leading to a straighter cross-section of the LAM. From the PC plots in Fig. 3b–d, it can be observed that the pessary descends into the LAM when moving from the supine to the upright position.

**Fig. 4:**
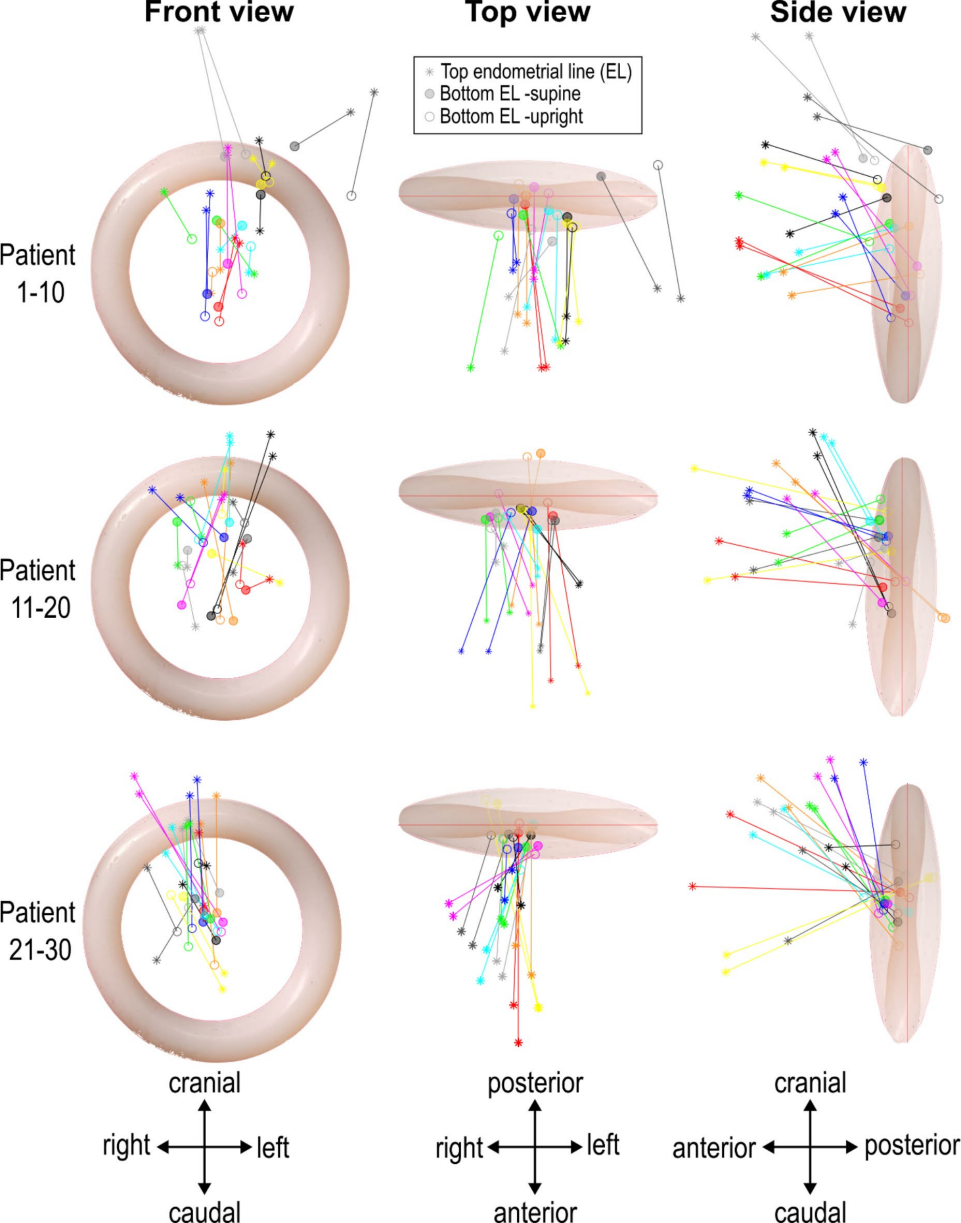
This figure illustrates the position of the endometrial line relative to the pessary in both supine and upright position for all patients (within rows, the same colours denote the same patient). To provide clarity, the figure presents front, top, and side views. Below each view, approximate coordinate directions are provided; however, these are not exact coordinates due to variations in the angle of the pessary among patients. Each row of the figure displays data for 10 patients.

The results of the analysis of the position of the uterus relative to the pessary are presented in Fig. 4. This figure indicates that the cervix is frequently positioned near or within the centre of the pessary, and that the movement of the uterus from the supine to the upright position is minimal, with the cervix remaining centrally located. However, there are a few exceptions; for instance, in one patient illustrated in grey in the top row of the figure, the uterus is resting on top of the pessary. It is important to note that these observations are relative to the pessary, and the angle of the pessary itself changes from supine to upright. Assessment of the position of the retroverted uteri showed a similar position to the anterior side of the pessary as the anteverted uteri.

## Discussion

This study was executed to gain a deeper understanding of the support mechanisms of ring pessaries and to evaluate the validity of several commonly held hypotheses regarding pessary support. Based on the position of the pessary in relation to bony landmarks, we can conclude that the bony structures do not play a direct role in pessary support. The position of the pubic symphysis is typically located above the lowest point of the pessary when women are in an upright position, as already hypothesized by Hong et al.^15^. In contrast, the LAM appears to play a crucial role in pessary support. As the body moves from a supine to an upright position, the pessary descends into the hammock-like structure of the LAM. For all women with a successful pessary fit, the LAM is consistently positioned significantly below the widest part of the pessary. Regarding the uterine lever hypothesis, our findings indicate that, in most of the pessary wearing POP population, the cervix is close to the centre of the pessary. The position of the cervix with respect to the pessary remains relatively stable between supine and upright position. Since, the pessary is slightly tilted within the body, the uterus likely pushes the pessary against the posterior vaginal wall, which may support the uterine lever hypothesis, even for a retroverted or retroflexed uterus.

Our upright analysis of the pessary allows us to define the position of a successfully fitted pessary within the body during normal daily activity. The pessary is located just inside the level of the introitus as described by Culligan^25^. On both lower sides the pessary are supported by the LAM. The lowest point of the pessary is likely not supported, at least not by the pubic symphysis, contrasting the previous hypothesis^10,11^. The cervix is close to the centre of the pessary for most patients. This means that the top of the pessary indeed often rests in the posterior fornix as has been suggested^11,15,17,26^. Since, the angles found in upright position are less than 90 degrees we expect that the uterus in most patients indeed acts as a lever pushing the pessary against the posterior vaginal wall. The interaction between the cervix, uterus and posterior vaginal wall deserves more attention in future research to study the reasons behind higher pessary dropout after hysterectomy^13,27^ and failure to relieve POP symptoms in patients with solitary predominant posterior compartment^3^.

The observation that the LAM provides support for the pessary may explain why LAM avulsions are associated with unsuccessful pessary fitting^3^, even though the degree of POP seems not always of influence^3,12,13,27^. We hypothesize that more advanced POP is likely to be present in women with avulsions, which compromises LAM support, thereby increasing the likelihood of an unsuccessful pessary fit, but advanced POP itself is not the reason for unsuccessful fitting. Additionally, Manzini et al.^14^ found that, especially in cases of avulsion (levator–urethra gap of ≥ 25 mm), the probability of pessary fitting success decrease with a larger hiatal area-to-ring pessary ratio during Valsalva manoeuvre. However, it remains challenging to determine which portions of the LAM are affected by damage and to what extent. Each of the pelvic floor muscles has a distinct line of action^28^, and damage to the different parts results in different functional impairments and pathological outcomes^29^. In this study, we assessed the entire LAM on a single imaging slice without distinguishing between the individual muscles. However, we demonstrated that LAM support is present in all women with a successful pessary fit included in our study. We hypothesize that the functional consequences of an avulsion affecting only a portion of the LAM can be compensated by the remaining intact parts somewhat keeping the integrity of the pelvic floor intact. Conversely, when damage extends to multiple LAM components, the integrity of the LAM may collapse to an extent that it can no longer support a conventional ring pessary. To test this hypothesis, it will be necessary to investigate LAM support in women who experience unsuccessful pessary fitting.

Our supine measurements of the lowest, mid-point and angle of the pessary closely align with the findings reported by Hong et al.^15^. Similarly, the lowest point values presented by Boogaard et al.^16^ are comparable to ours. However, their angle measurements show significantly different results, which may be attributed to the large variation in angle across patients observed in all three studies, as well as the smaller sample size in their study (9 patients, compared to 21 by Hong et al.^15^ and 30 in the present study). This considerable variation found in all three studies between patients should also keep us away from drawing to strong conclusions on the significant difference in angle found in this study between supine and upright. It is worth noting that the movement Boogaard et al.^16^ observe from rest to Valsalva is smaller than the movement we observe from supine to upright, highlighting the importance of assessing the pelvic floor and its interaction with pessary in upright position, which more accurately reflects daily functional activity.

The primary strength of this study lies in our analysis of pessary position using MRI with patients in an upright position. This approach more accurately reflects the forces acting on the pessary during normal daily activities. Consequently, it allows for a more reliable investigation of pessary support mechanisms. Another strength is the application of PCA, this allows for comparing data form a diverse population with different hiatal and pessary size and find the most prominent shape factors, while also allowing for statistical analysis of these factors. This allowed us to show that the pessary for all women is farther away from the bottom of the LAM and that it descends with respect to the LAM in upright position.

One of the limitations of our study is that upright MRI is not widely available in clinical practice. We consider this work to be clinically fundamental research aimed at enhancing our understanding of pessary support. Upright imaging has proven effective in analysing pelvic floor disorders^18–20^ and we hope that this study can serve as a call to the urogynaecology community to explore ways to make upright imaging accessible in clinical practice, whether through upright MRI or exploring alternative methods for acquiring pelvic floor ultrasound data in upright position, since the integrity of the LAM can also be investigated on ultrasound^30^. Our conclusion on the support of the LAM should only be considered proven in patients with primarily anterior and/or apical POP. Since this will be the majority of the patients using a pessary, the outcome of the study can still be considered relatively generalizable.

The part of the LAM which is shown and assessed in the cross section of the pessary is different for upright and supine images. This, however, does not limit our conclusion that from supine towards upright, the pessary rotates and descends into the hammock-like structure that is the LAM. We observe in our PCA that the LAM comes closer to the pessary. However, we should be careful on the observed difference in length of the LAM since we are looking at different cross sections of the LAM. In future research this might be avoided by generating a 3D model of the LAM for all patients.

Future research to reinforce our findings should focus on investigating patients with unsuccessful pessary fittings using upright MRI. This will allow for a comparison to determine if the support mechanisms identified in this study are absent in patients with an unsuccessful fit and to identify any additional pessary support mechanisms. A second line of interest could be the investigation of non-ring shape pessaries (e.g. Gellhorn, donut and cube pessaries), since their support mechanisms are different. Thirdly, we only investigated pessary support, since lack of support is expected to cause pessary dislodgement, however other reasons to stop pessary treatment, like excessive vaginal discharge or pain, should be investigated as well. Finally, another key area of investigation is the effect of pessary placement on pelvic floor structures itself, such as the LAM, bladder, and uterus. Meaning, that we need to investigate how the pelvic structures displace and deform based on the pessary insertion and how this is related to PFD complaints.

In conclusion, we showed that the position of a pessary in the pelvis differs significantly between supine and upright position. From our upright analysis of the pessary position we can conclude that the pubic bones do not play a direct role in pessary support. However, the levator ani muscles play a significant role in pessary support. Also, the uterus is likely to add to the pessary support by pushing it to the posterior vaginal wall.

## Data Availability

The patient data used in this study is not publicly available to preserve individuals’ privacy under the European General Data Protection Regulation. The first or last author can be contacted, regarding questions on the data or sharing of anonymized measurements.
